# Predicting Knee Osteoarthritis Severity from Radiographic Predictors: Data from the Osteoarthritis Initiative

**DOI:** 10.1007/s10439-025-03740-z

**Published:** 2025-05-09

**Authors:** Teemu A. T. Nurmirinta, Mikael J. Turunen, Jussi Tohka, Mika E. Mononen, Mimmi K. Liukkonen

**Affiliations:** 1 Department of Technical Physics, University of Eastern Finland, Kuopio, Finland; 2 Diagnostic Imaging Centre, Kuopio University Hospital, The Wellbeing Services County of North Savo, Kuopio, Finland; 3 Science Service Center, Kuopio University Hospital, The Wellbeing Services County of North Savo, Kuopio, Finland; 4 AI Virtanen Institute for Molecular Sciences, University of Eastern Finland, Kuopio, Finland

**Keywords:** Machine learning, X-ray, Multiclass prediction, Severity, OAI, Imaging

## Abstract

**Purpose:**

In knee osteoarthritis (KOA) treatment, preventive measures to reduce its onset risk are a key factor. Among individuals with radiographically healthy knees, however, future knee joint integrity and condition cannot be predicted by clinically applicable methods. We investigated if knee joint morphology derived from widely accessible and cost-effective radiographs could be helpful in predicting future knee joint integrity and condition.

**Methods:**

We combined knee joint morphology with known risk predictors such as age, height, and weight. Baseline data were utilized as predictors, and the maximal severity of KOA after 8 years served as a target variable. The three KOA categories in this study were based on Kellgren–Lawrence grading: healthy, moderate, and severe. We employed a two-stage machine learning model that utilized two random forest algorithms. We trained three models: the subject demographics (SD) model utilized only SD; the image model utilized only knee joint morphology from radiographs; the merged model utilized combined predictors. The training data comprised an 8-year follow-up of 1222 knees from 683 individuals.

**Results:**

The SD- model obtained a weighted F1 score (WF1) of 77.2% and a balanced accuracy (BA) of 65.6%. The Image-model performance metrics were lowest, with a WF1 of 76.5% and BA of 63.8%. The top-performing merged model achieved a WF1 score of 78.3% and a BA of 68.2%.

**Conclusion:**

Our two-stage prediction model provided improved results based on performance metrics, suggesting potential for application in clinical settings.

**Supplementary Information:**

The online version contains supplementary material available at 10.1007/s10439-025-03740-z.

## Introduction

Knee osteoarthritis (KOA) is a common pathological condition characterized by the degeneration of the knee joint. Management of the KOA is extremely challenging since there is no cure for the disease, and it usually progresses to a stage of unbearable pain leading to a total knee replacement surgery (TKR), which is considered the only sustainable solution for pain relief [1]. However, this kind of reactive KOA management is very expensive. Therefore, proactive solutions are needed. Preventative treatments, such as physical therapy, could be targeted to individuals who are at high risk of developing KOA [2]. Prediction could also be used for motivating overweight individuals to lose weight. The primary reason for the lack of proactive methods in KOA management is the absence of solutions to predict future knee health in clinical practice.

Several machine learning (ML)-based KOA prediction methods have been proposed in the literature [3–10]. Most of these studies focus on binary prediction (No KOA vs. KOA), which is easier than multiclass prediction. However, the capability to provide multiple KOA severity classes would allow more personalized treatment planning. A recent multiclass prediction study [8] predicted KOA progression into four different classes: one non-progressive and three progressive classes. The main limitation of the study was that developed model requires at least a 2-year clinical trial to observe all predictors (changes in knee joint space width). Additionally, pain questionnaires are highly subjective and thus not comparable between different subjects. Therefore, there is a need for models that use only objective and less error-prone baseline predictors that need to be measured only once.

The growing amount of KOA data has allowed researchers to test a wide range of predictors for KOA prediction [7, 9]. A recent study [9] tested 112 different predictors with high variety and trained a second model with 10 predictors that had the highest importance. The 112-predictor model AUC value (0.792) was not significantly higher than the 10-predictor model (AUC 0.772). Increasing the number of predictors might slightly increase model performance, but performing multiple different tests to get the needed data for KOA prediction is not feasible in clinical settings. An optimal model would use a reasonable number of predictors that are easy and fast to collect to keep the duration of the clinical visit tolerable. One possible solution would be using physical measures that can be quantified objectively based on one clinical visit and not questionnaires that are inherently subjective.

In our previous study [11], we used a novel two-stage prediction model and knee joint dimensions obtained from magnetic resonance imaging (MRI) for predicting KOA. The results showed that the two-stage prediction model outperforms the traditional single prediction algorithm when predicting KOA into multiple grades with an imbalanced class distribution. The limitation of the developed method was that MRI-based prediction models are not suitable in primary healthcare due to poor availability, high price, and limited time requirements. One solution could be using radiographs, which are less expensive than an MRI and are mostly prescribed before MRI, especially in primary healthcare. Radiographs are superior at visualizing bony structures, making them useful for diagnosing joint space narrowing, bone spurs, bone alignment, and structural anomalies.

In this study, we aimed to train a cost-effective machine learning multiclass prediction model to predict the onset of KOA based on subject demographics and quantified measures from the standard anteroposterior (AP) knee radiographs. We hypothesized that these measures from knee radiographs could provide a cost-effective and accurate alternative to MRI-based predictions and therefore, provide a simple technique to be utilized in primary healthcare. Our prediction is based on the two-stage ML model [11] and knee joint morphology derived from radiographs. If easily measured parameters can be used for building an accurate and cost-effective prognostic model, it will provide a new, straightforward technique for predicting KOA in the future and improving patient care.

## Methods

### Osteoarthritis Initiative Database

We used data from the Osteoarthritis Initiative (OAI, https://nda.nih.gov/oai) database in this study. Bilateral anteroposterior fixed flexion knee radiographs were acquired in accordance with typical guidelines for annual and total radiation dosage for research subjects. Written consent was obtained from all subjects prior to each clinic visit. The OAI study was approved by the Institutional Review Board for the University of California, San Francisco, and its affiliates. The IRB approval was also obtained from all four clinical sites located at Brown University in Rhode Island, Ohio State University in Columbus, Ohio, the University of Maryland/Johns Hopkins University joint center in Baltimore, Maryland, and the University of Pittsburgh in Pennsylvania. Further details about the OAI data are accessible on the OAI website (https://nda.nih.gov/oai/).

### Exclusion Criteria

**Fig. 1:**
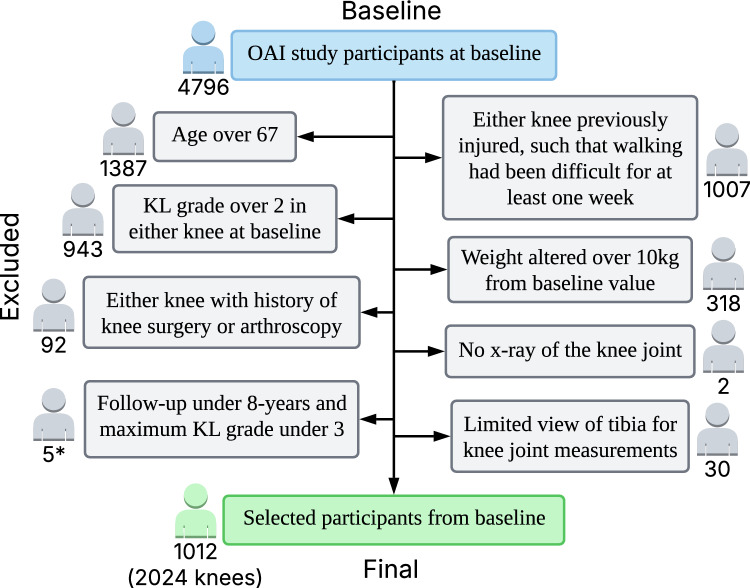
Flowchart of excluded participants for machine learning model development and evaluation. *The number of knees with a follow-up of less than 8 years that were included because KOA had already progressed to KL3 or higher was 143

We were interested in studying a healthy working age population who are at risk for the onset of knee osteoarthritis. Therefore, we excluded participants who had a Kellgren–Lawrence [12] (KL) grade 2 or over in either knee at baseline and we set the age range from 45 to 67 years old. Our focus was on predicting primary KOA, and thus participants who had either knee previously injured, such that walking had been difficult for at least 1 week or had previously undergone knee surgery were excluded from the study. Those participant whose weight differed more than 10 kg from the baseline at any point during the follow-up (1, 2, 4, 6, 8 years) were excluded. This was because we only used baseline weight value and changes in weight could have unpredictable outcomes. If a participant was followed up less than 8 years, they were excluded unless KOA had already progressed to KL3 or higher. Because it is unlikely that the progression of the KOA would reverse, therefore after 8 years, these individuals KOA class will be KL3 or above (severe). For example, if participant follow-up data was not complete but right knee KL grade was 3 after 4 years, right knee was included to increase the number of participants with higher KL grades. The number of knees that were included because KOA had already progressed to KL3 or higher was 143. This also means that for some participants only one knee was included. Participants without knee joint x-rays or with limited views of the tibia were excluded because knee joint analysis was not possible. The OAI data contained data from 4796 participants, of whom 683 (1222 knees) were eligible for our further analyses (see Fig. 1).

### Model Predictors and Targets

**Fig. 2:**
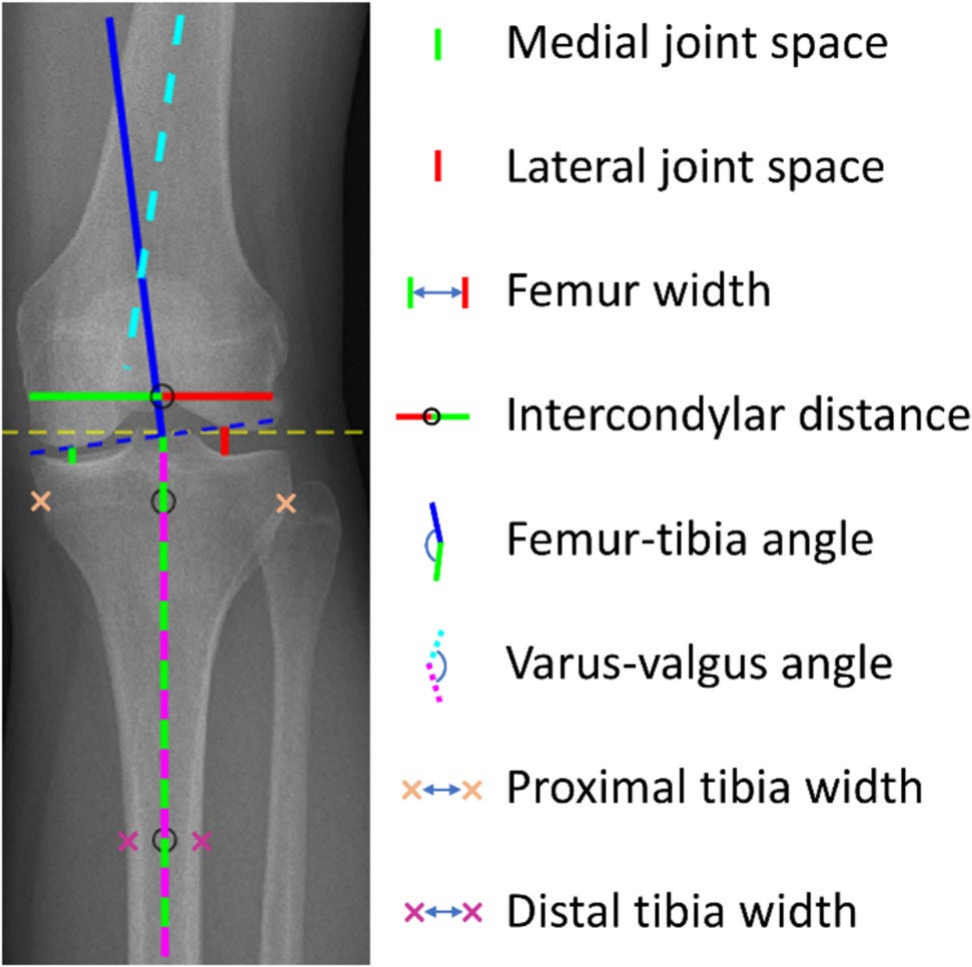
Knee dimensions and angles measured from the anteroposterior knee radiographs using a semi-automatic, custom-made MATLAB (The MathWorks Inc. MATLAB R2023b v.23.2) graphical user interface (GUI)-based application [12]. The GUI initially marked each leg in the radiograph as left and right knee and identified the location of joint spaces with yellow dashed lines. The GUI used vertical and lateral intensity profile estimations to approximate the locations of the 2-dimensional coordinates necessary for dimension and angle measurements. After the automated stage, a single experienced user manually adjusted or verified every set coordinate for each knee. The GUI displayed a pop-up window with region magnification to help users position the measurement coordinates accurately

We selected 12 predictors (Table 1) from the OAI database. These predictors were age, height, weight, baseline KL grade, and eight image-based predictors measured from anteroposterior knee radiographs. The image-based predictors were medial side joint space, lateral side joint space, femur width, intercondylar distance, angle between femur and tibia (FT angle [13]), angle between the anatomic axes of the femur and tibia (varus-valgus angle), proximal tibia width, and distal tibia width shown in Fig. 2. Image-based predictors were measured using previously developed semi-automatic MATLAB (The MathWorks Inc. MATLAB R2023b v.23.2) script [14]. Measuring takes around 1 to 4 min per image (two knees). Prediction targets were based on the maximal KL grades after 8-year follow-up (baseline KL < 2), which were re-categorized into three classes:

- KL01: KL grades 0 and 1, representing healthy knees (950 knees, 77.7% of total)
- KL2: KL grade 2, representing moderate KOA (140 knees, 11.5% of total)
- KL34: KL grades 3, 4, and total knee replacement, representing severe KOA (132 knees, 10.8% of total)

**Table 1:** Category, number, description, computed mean, and standard deviation (SD) of each predictor

Category	#	Predictors: description	Mean ± SD
Demographics	1	Age: Age at start of evaluation (45–67 years)	56.7 ± 6.1 years
	2	Height: Average height	165.1 ± 20.0 mm
	3	Weight: Average weight on scale	76.2 ± 15.7 kg
Knee joint measures from radiographs (Image predictors) (see Fig. 2)	4	Medial JS: Medial side joint space	4.7 ± 0.9 mm
	5	Lateral JS: Lateral side joint space	6.7 ± 1.2 mm
	6	Femur width: Total width of the condyles at the top of the intercondylar fossa	76.5 ± 7.0 mm
	7	Intercondylar distance: Distance between Medial and Lateral JS centerlines	48.8 ± 4.8 mm
	8	FT-angle: Angle (°) between femur and tibia	− 6.7 ± 1.8⁰
	9	VV-angle: The angle between the anatomic axes of the femur and tibia	4.6 ± 3.0⁰
	10	Proximal tibia width: Proximal tibia width measured 1 cm from the tibial plateau	77.0 ± 7.1 mm
	11	Distal tibia width: Distal tibia width measured 10 cm from the tibial plateau	27.4 ± 4.1 mm
Baseline KL grade	12	KL grade: Kellgren–Lawrence (KL) grade at baseline evaluation	0.18 ± 0.57
Target	–	KL max: Maximum KL grade in 8-year evaluation	0.66 ± 1.3

The angle “⁰” is in degrees

### Prediction Model

**Fig. 3:**
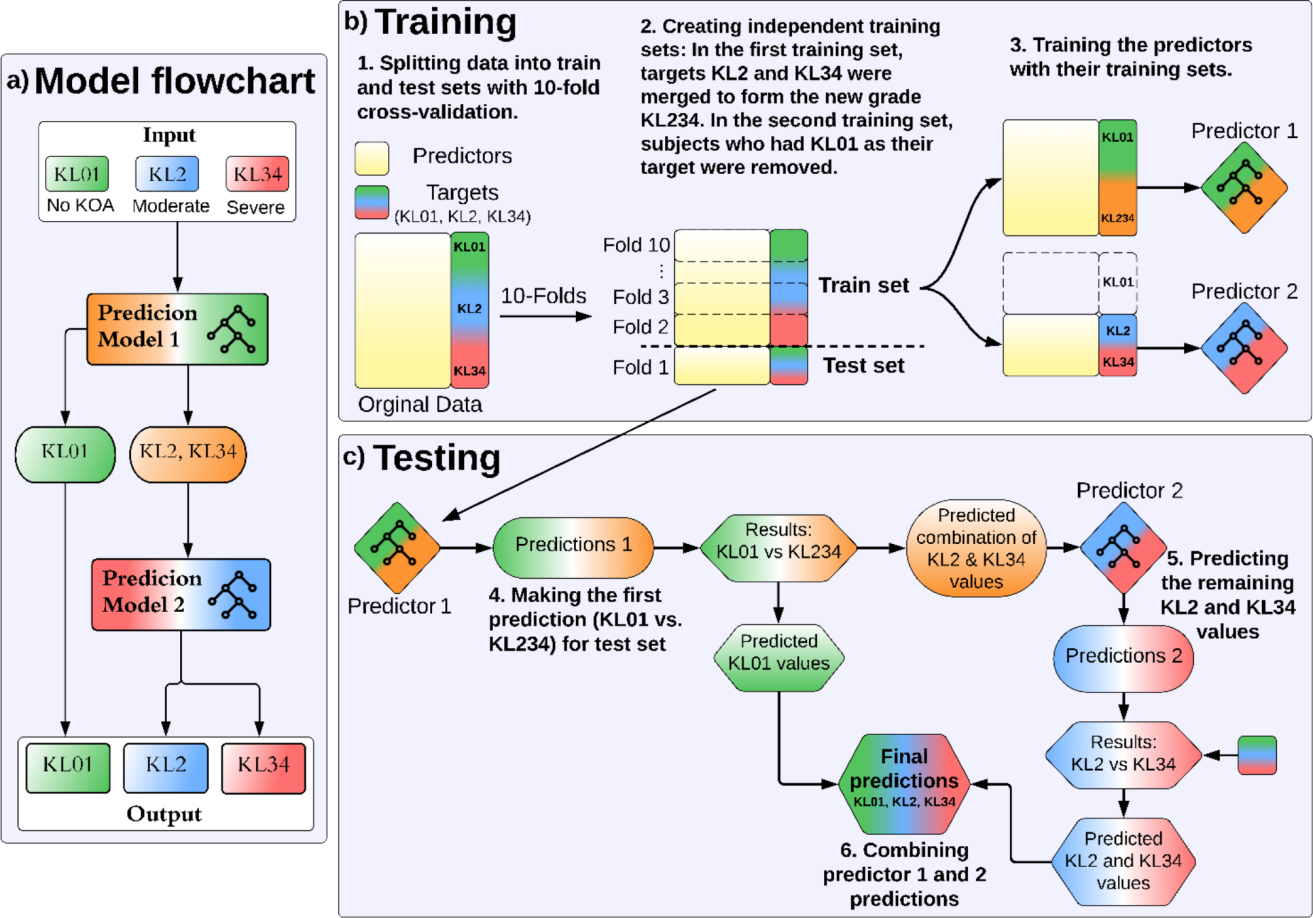
**a** Flowchart of a two-stage prediction model. KL01 and a combination of KL2 and KL34 are predicted by the first-stage prediction model. The predicted KL01 values are output directly, while the KL2 and KL34 values are passed into the second-stage prediction model, which predicts them into separate groups. **b** Flowchart of cross-validation training and **c** testing for each fold. During 10-fold cross-validation, the training and testing procedures are iterated 10 times. Each iteration involves using one fold as the test set and the remaining folds as the training set

ML prediction was based on a two-stage prediction model, introduced in our previous study [11]. This method utilizes KL-grading ordinality to split the difficult multiclass prediction tasks into two binary prediction tasks. The first binary prediction (first stage) uses an ML algorithm to predict KL01 and the combined KL2 and KL34 group. If the prediction in the first stage is KL01, it is assigned as the final prediction result and there is no second-stage prediction. If the prediction in the first stage is the combined KL2 and KL34 group, we continue to the second prediction algorithm (second stage). The second-stage prediction distinguishes KL2 and KL34 and the output of the second-stage prediction (KL2 or KL34) is assigned as the final prediction result. Model prediction flowchart is shown in Fig. 3a and more detailed training and testing flowcharts in Fig. 3b and c.

As we showed in our previous study [11], when predicting KOA into multiple unevenly distributed classes, the two-stage prediction model outperforms the traditional single KOA prediction technique. We also tested several ML algorithms for prediction, and the imbalanced-learn library’s Balanced Random Forest (BRF) [15] performed the best [11]. During hyperparameter tuning, we discovered that increasing the number of trees in the forest beyond 3000 did not result in notable improvements in performance (see Fig. S1). Therefore, we used 3000 trees in both stages. Other hyperparameters were unchanged. For evaluation, we used Stratified 10-fold Cross-Validation (CV) [16] with the constraint that the different knees of the same person were always in the same fold, thus eliminating the possibility that the data from a single person would be divided between train and test sets. The stratified 10-fold CV splits the dataset into ten equal subsets, ensuring that the class distribution in each fold corresponds to the overall class distribution of the dataset. Ninefolds are used for training and one for validation. This procedure is repeated ten times to evaluate the model's overall performance. Model training was done using Python (v. 3.9.7), Jupyter Notebook (v. 6.4.5), the scikit-learn library (v. 1.2.2), and imbalanced-learn library (v. 0.12.3).

### Experiments

We were interested in testing if knee morphology (image predictors) obtained from radiographs could improve the prediction of KOA when combined with subject demographics (age, height, and weight). Therefore, we trained three different models:

- SD-model with subject demographics (SD) predictors (1–3, and 12 in Table 1)
- Image-model with knee joint morphological predictors from radiographic images (4–12 in Table 1)
- Merged-model with a combination of demographic and knee joint morphological predictors (1–12 in Table 1).

### Model Predictor Analysis

**Fig. 4:**
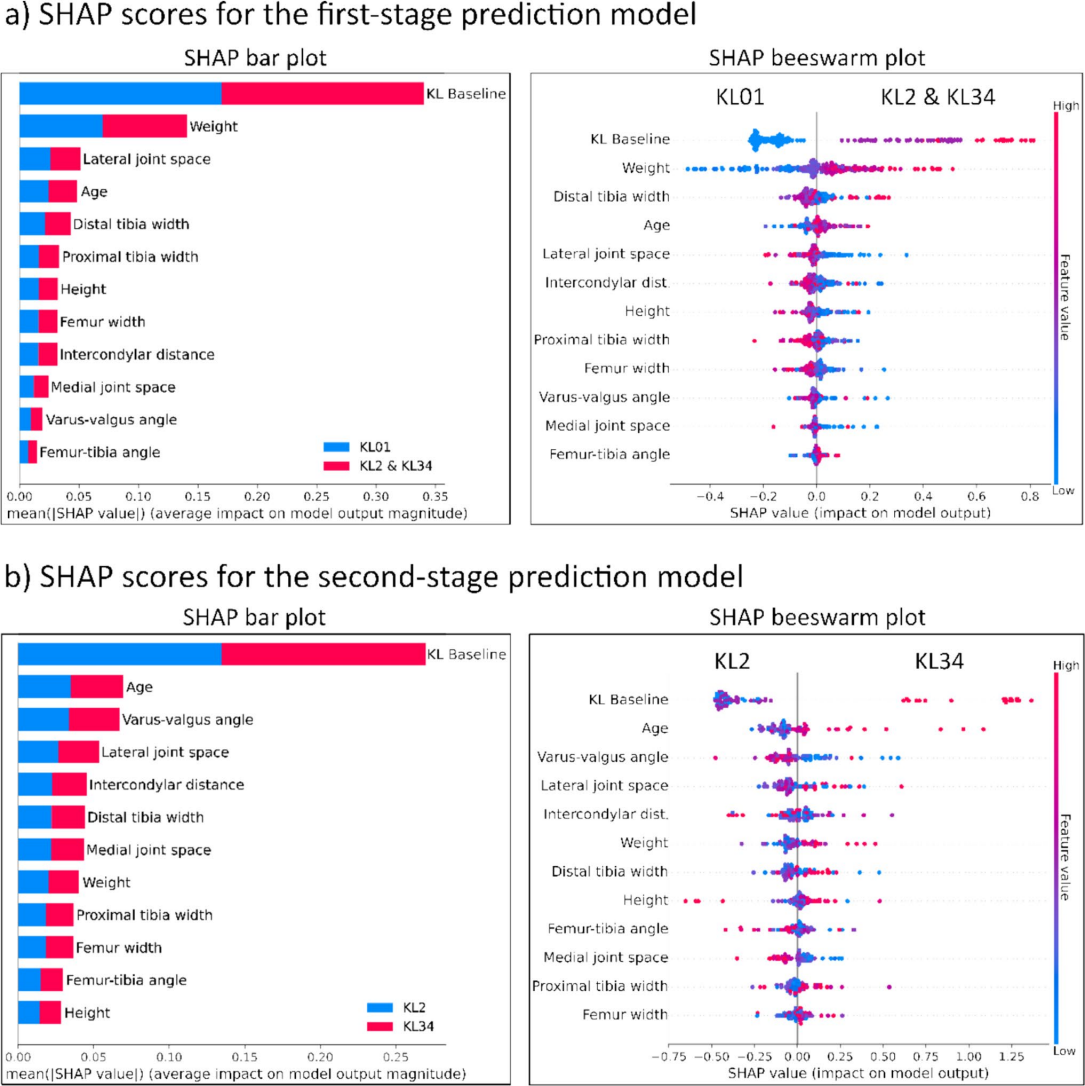
Shapley additive explanations (SHAP) scores for each predictor in **a** the first stage and **b** the second-stage classifications of Merged-Model. The SHAP bar plot on the left side shows the mean absolute SHAP values for each predictor, with higher bars indicating predictors that have a greater influence on the output of the model. Different colors indicate the impact on various targets (KL grades). On the right side, the SHAP beeswarm plot displays the impact of each predictor on the prediction of the model. Predictors are listed on the *y*-axis, sorted by importance. Each dot represents a SHAP value for a predictor in a specific instance, with color indicating predictor value (e.g., red for high, blue for low). The density of the points along the *x*-axis illustrates the distribution of SHAP values for each predictor, highlighting variability and outliers. Point placement on the *x*-axis tells the impact on the prediction of the model. Example on a) SHAP beeswarm plot lower weights (blue) on the left side indicate KL01, while higher weights (red) on the right side indicate KL2 and KL34

To analyze and illustrate contributions of individual predictors to the performance of the model, we made SHapley Additive ExPlanations (SHAP) [17] bar and beeswarm plots for the Merged-model (Fig. 4). SHAP analysis is an excellent tool for investigating the effects of individual predictors on prediction. However, if predictors are correlated, SHAP values may be skewed [18]. Therefore, we added a correlation matrix to supplementary (Fig. S2). SHAP analysis was done using Python (v. 3.9.7) and the shap library. Correlation analysis was performed and visualized in R (v. 4.3.1) using the corrplot and Hmisc libraries.

### Performance Evaluation Methods and Statistical Analysis

The performance of the models was assessed through a stratified 10-fold CV. To reduce the variance due to different train/test divisions, we repeated 10-fold CV 25 times and reported the average performance measures across 25 repeats [8]. As evaluation metrics, we utilized Balanced Accuracy (BA) [19], and Weighted F1 (WF1) [20] metrics. We used the WF1 score because it accounts for imbalances in class distribution. The F1 score has the disadvantage of not considering true negatives. To account for true negatives, we computed BA score [21]. BA takes imbalanced class distributions better into account and provides a more correct representation of the prediction model performance than overall accuracy [19, 22].

Statistical analysis between two models was conducted using a corrected repeated k-fold CV test [23]. The test statistic, distributed according to a t-distribution with $\text{d}f= kr-1$ degrees of freedom, is defined as

$$t= \frac{\frac{1}{k*r}{\sum }_{i=0}^{k}{\sum }_{j=0}^{r}{x}_{ij}}{\sqrt{\left(\frac{1}{k*r}+ \frac{{n}_{2}}{{n}_{1}}\right){\widehat{\sigma }}^{2}}}$$

where *n*_1_ is the number of instances used for training, and *n*_2_ is the number of instances used for testing, ${x}_{ij}={a}_{ij}-{b}_{ij}$ is the difference between model *a* and *b* for fold *i* and run *j*, *r* is the number of CV repeats, and *k* is the number of folds. ${\widehat{\sigma }}^{2}$ is estimate of variance, and it is calculated as follows

$${\widehat{\sigma }}^{2}= \frac{1}{k*r-1}\sum\limits_{i=1}^{k}\sum\limits_{j=1}^{r}{(x}_{ij}-m)^2$$

where $m= \frac{1}{k*r-1}{\sum }_{i=1}^{k}{\sum }_{j=1}^{r}{x}_{ij}$ is an estimate of the mean. In our statistical analysis, we set the significance level (alpha) at 0.01 to determine statistical significance. All hypothesis tests were conducted as two-tailed tests.

## Results

For the first-stage prediction, the SHAP bar plot (Fig. 4a) clearly shows that KL baseline and weight were the most important predictors. Following that came lateral joint space, age, and distal tibia width predictors. The SHAP beeswarm plot provides similar results, but distal tibia width is recognized as a more important predictor than age and lateral joint space. The varus-valgus and femur-tibia angles were the least important predictors during the first stage. For the second-stage prediction SHAP bar plot reveals that the KL baseline was the most important predictor. Interestingly, weight, which was very important in the first stage, became less important in the second stage. The varus-valgus angle had the reverse impact, being less important in the first stage but more important in the second stage. The medial joint space appears to be less important than the lateral side in both stages. The second-stage SHAP beeswarm reveals that higher KL baseline, age, weight, and smaller varus-valgus angle value (varus alignment) indicate severe KOA (KL 34).

**Fig. 5:**
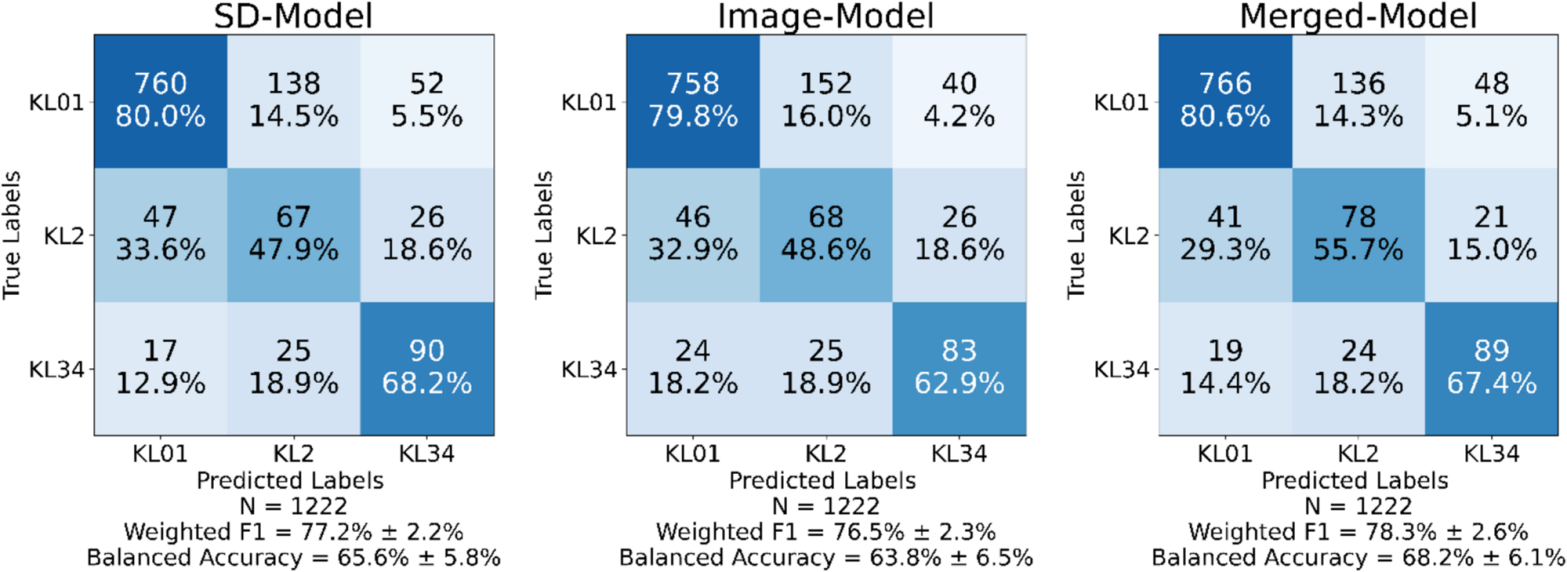
Confusion matrices for SD-Model trained on subject demographic (SD) predictors (1–3, 12 in Table 1), Image-Model trained on knee joint morphological predictors (4–12 in Table 1) from radiographs, and Merged-Model trained on a combination of demographic and knee joint morphological predictors (1–12 in Table 1)

The Merged-model, which combines predictors from SD and Image-models, achieved the highest WF1 (78.3 ± 2.6%) and BA (68.2 ± 6.1%) scores among the three models examined. The Merged model achieved the highest KL01 (80.6%) grade sensitivity, with only 5.1% of KL01 grades mistakenly predicted to KL34. Merged-model had a 7.8% greater sensitivity of KL2 (55.7%) grade than the SD model. Higher KL2 grade sensitivity could be useful for health professionals who may obtain more detailed information on KOA development. The SD-model achieved 77.2 ± 2.2% WF1 and 65.6 ± 5.8% BA, placing it as the second best. The SD-model predicted KL34 grade with accuracy of 68.2%. The Image-model had the lowest performance metrics (WF1 of 76.5 ± 2.3% and BA of 63.8 ± 6.5%). Figure 5 shows each model confusion matrix, along with performance meters for WF1 and BA scores.

Statistical analysis showed that the Merged-model had significantly higher (*p* < 0.00001) performance scores than the SD, and Image-model. Also, SD-model had significantly higher WF1, and BA scores (*p* < 0.00001) than the Image-model.

## Discussion

In this study, we trained a machine learning-based model for predicting future knee joint integrity and condition. We used a two-stage prediction model [11] based on knee joint morphology derived from widely available and inexpensive radiographs to improve the difficult process of predicting KOA into multiple severity grades. Overall, our two-stage technique resulted in a WF1 prediction score of 76.5 to 78.3% and BA values of 63.8 to 68.2%.

The SHAP beeswarm plot for the first-stage prediction model (Fig. 4a) shows that weight is clearly separated so that lower weight (blue dots) indicates KL01 and greater weight (red dots) KL234. This is expected given that overweight and obesity are recognized to be significant risk factors for KOA [24]. In the second prediction model (Fig. 4b), weight SHAP values were closer together, except for four higher weight participants that indicate KL34. The varus-valgus angle was insignificant in the first stage, but it became the third most important predictor in the second stage. In the second stage, a lower varus-valgus angle (varus alignment) predicts a greater likelihood of severe KOA. A varus alignment has been demonstrated to accelerate medial side KOA development and is associated with joint space reduction [25]. The joint space narrowing is more prevalent in the medial than in the lateral side [26]. As a result, it is surprising to see that the medial joint space appears to be less significant in the prediction than the lateral side at both stages. Since cartilage thickness is generally greater on the lateral side, changes in thickness in this side may serve as a more sensitive predictive parameter. Conversely, on the medial side, thinning may reach a saturation point where only minimal cartilage remains, reducing the sensitivity of thickness changes as a predictive marker.

Most studies utilize only binary prediction models (No KOA vs. KOA) and measure their performance using the area under the receiver operating characteristic curve (AUC) [9, 27, 28], so comparing our models to previous ones is challenging. Even when a model performs poorly in minority classes, AUC might provide good results; thus, for evaluating performance on multiclass imbalanced datasets, it is not the best method. Current state-of-the-art multiclass techniques, with a WF1 score of 69.0% [8], are outperformed by our proposed two-stage model with 78.3%. Notably, between our two studies, datasets and sizes differed. In our earlier study [11], we employed the same two-stage method; instead of a radiograph, however, we used an MRI. The MRI-based model (Model 2KL) in that study achieved 79.0% WF1 and 65.7% BA, and the radiograph-based one in our current study achieved 78.3% WF1 and 68.2% BA. Model 2KL exhibited 0.7% higher WF1 and 2.5% lower BA. The reason could be that, due to different imaging methods and participant exclusion criteria, in our present trial, certain participants were different. SD-models lack knee morphology information. When we compare our SD-model to the corresponding model (Model 1KL) in the first study, we observe that Model 1KL performed better. In Model 1KL, WF1 was 77.8% and BA 64.9%; in the SD-model, WF1 was 75.7% and BA 61.4%, implying that the current study subjects may be more difficult to predict. To identify the reason behind these differences in values, we must evaluate these models, using external datasets.

Our method has limitations; we did not validate our prediction model against an external dataset; our estimate of model performance could thus be biased. We aimed to exclude individuals who had previously experienced knee injuries since injuries are difficult to predict. The participants were excluded based on pain (walking had been difficult for at least 1 week); this leaves the possibility of some minor cartilage injury that did not cause pain. Detecting minor injuries would most likely require the use of MRI imaging. In our current model, knee joint dimension measurements take around 1 to 4 min; depending on the person measuring, dimensions might vary. Using deep learning methods utilizing key point detection algorithms, however, measurements could be automated in the future.”

Overall, our results demonstrated that knee joint dimensions and angles provide good prediction performance with radiographic images; therefore, for this purpose, an MRI is unnecessary. With scalability, our model would provide a new and straightforward technique for predicting future knee integrity and condition, improving current KOA management strategies via prevention. A scalable approach that matches any imaging modality that accurately identifies the bone would enable quantitative tools to evaluate personalized risk for (future) KOA development. It would also allow visualizing the effects of preventive measures, such as weight loss; such visualization can be important for patient motivation or engagement [29].

## Supplementary Information

Below is the link to the electronic supplementary material.

Supplementary file1 (DOCX 322 kb)

## Data Availability

Information regarding OAI data availability can be found on the OAI website (https://nda.nih.gov/oai/). Data-related knee joint dimensions measured from OAI data X-rays with ID information can be shared upon reasonable request.

## References

[1] Neogi, T. The epidemiology and impact of pain in osteoarthritis. *Osteoarthr. Cartil.* 21(9):1145–1153, 2013. 10.1016/j.joca.2013.03.018. 10.1016/j.joca.2013.03.018 PMC3753584 23973124

[2] Mononen, M. E., M. J. Turunen, L. Stenroth, S. Saarakkala, and M. Boesen. Biomechanical modeling and imaging for knee osteoarthritis—is there a role for AI? *Osteoarthr. Imaging*. 4(2):100182, 2024. 10.1016/j.ostima.2024.100182. 10.1016/j.ostima.2024.100182 PMC13228678 42238770

[3] Brahim, A., et al. A decision support tool for early detection of knee OsteoArthritis using X-ray imaging and machine learning: data from the OsteoArthritis Initiative. *Comput. Med. Imaging Graph.* 73:11–18, 2019. 10.1016/j.compmedimag.2019.01.007. 30784984 10.1016/j.compmedimag.2019.01.007

[4] Jamshidi, A., J.-P. Pelletier, and J. Martel-Pelletier. Machine-learning-based patient-specific prediction models for knee osteoarthritis. *Nat. Rev. Rheumatol.* 15(1):49–60, 2019. 10.1038/s41584-018-0130-5. 30523334 10.1038/s41584-018-0130-5

[5] Kokkotis, C., S. Moustakidis, E. Papageorgiou, G. Giakas, and D. E. Tsaopoulos. Machine learning in knee osteoarthritis: a review. *Osteoarthr. Cartil. Open*. 2(3):100069–100069, 2020. 10.1016/j.ocarto.2020.100069. 36474688 10.1016/j.ocarto.2020.100069 PMC9718265

[6] Tiulpin, A., et al. Multimodal machine learning-based knee osteoarthritis progression prediction from plain radiographs and clinical data. *Sci. Rep.* 9(1):20038–20111, 2019. 10.1038/s41598-019-56527-3. 31882803 10.1038/s41598-019-56527-3 PMC6934728

[7] Tolpadi, A. A., J. J. Lee, V. Pedoia, and S. Majumdar. Deep learning predicts total knee replacement from magnetic resonance images. *Sci. Rep.* 10(1):6371–6371, 2020. 10.1038/s41598-020-63395-9. 32286452 10.1038/s41598-020-63395-9 PMC7156761

[8] Widera, P., et al. Multi-classifier prediction of knee osteoarthritis progression from incomplete imbalanced longitudinal data. *Sci. Rep.* 10(1):8427–8427, 2020. 10.1038/s41598-020-64643-8. 32439879 10.1038/s41598-020-64643-8 PMC7242357

[9] Joseph, G. B., C. E. McCulloch, M. C. Nevitt, T. M. Link, and J. H. Sohn. Machine learning to predict incident radiographic knee osteoarthritis over 8 years using combined MR imaging features, demographics, and clinical factors: data from the Osteoarthritis Initiative. *Osteoarthr. Cartil.* 30(2):270–279, 2022. 10.1016/j.joca.2021.11.007. 10.1016/j.joca.2021.11.007 PMC8792367 34800631

[10] Nair, A., M. A. Alagha, J. Cobb, and G. Jones. Assessing the value of imaging data in machine learning models to predict patient-reported outcome measures in knee osteoarthritis patients. *Bioengineering*. 11(8):824, 2024. 10.3390/bioengineering11080824. 39199782 10.3390/bioengineering11080824 PMC11351307

[11] Nurmirinta, T. A. T., M. J. Turunen, R. K. Korhonen, J. Tohka, M. K. Liukkonen, and M. E. Mononen. Two-stage classification of future knee osteoarthritis severity after 8 years using MRI: data from the Osteoarthritis Initiative. *Ann. Biomed. Eng.* 2024. 10.1007/s10439-024-03578-x. 38980544 10.1007/s10439-024-03578-x PMC11560993

[12] Kellgren, J. H., and J. S. Lawrence. Radiological assessment of osteo-arthrosis. *Ann. Rheum. Dis.* 16(4):494–502, 1957. 10.1136/ard.16.4.494. 13498604 10.1136/ard.16.4.494 PMC1006995

[13] Iranpour-Boroujeni, T., J. Li, J. A. Lynch, M. Nevitt, and J. Duryea. A new method to measure anatomic knee alignment for large studies of OA: data from the Osteoarthritis Initiative. *Osteoarthr. Cartil.* 22(10):1668–1674, 2014. 10.1016/j.joca.2014.06.011. 10.1016/j.joca.2014.06.011 25278076

[14] Mononen, M. E., M. K. Liukkonen, and M. J. Turunen. X-ray with finite element analysis is a viable alternative for MRI to predict knee osteoarthritis: data from the Osteoarthritis Initiative. *J. Orthop. Res.* 42(9):1964–1973, 2024. 10.1002/jor.25861. 38650428 10.1002/jor.25861

[15] Chen, C., A. Liaw, and L. Breiman. Using Random Forest to Learn Imbalanced Data. Berkeley: University of California, 2005.

[16] Pedregosa, F., et al. Scikit-learn: machine learning in Python. *J. Mach. Learn. Res.* 12:2825–2830, 2011. 10.5555/1953048.2078195.

[17] Lundberg, S., and S.-I. Lee. A unified approach to interpreting model predictions. 2017. arXiv.org. 10.48550/arxiv.1705.07874.

[18] Aas, K., M. Jullum, and A. Løland. Explaining individual predictions when features are dependent: more accurate approximations to Shapley values. *Artif. Intell.* 298:103502, 2021. 10.1016/j.artint.2021.103502.

[19] Kubat, M., and S. Matwin. Addressing the curse of imbalanced training sets: one-sided selection. In: Fourteenth International Conference on Machine Learning, 1997.

[20] Chinchor, N. MUC-4 evaluation metrics. In: Proceedings of the 4th Conference on Message Understanding—MUC4 ’92. San Diego, USA: Association for Computational Linguistics, 1992, pp. 22–29. 10.3115/1072064.1072067.

[21] Powers, R., M. Goldszmidt, and I. Cohen. Short term performance forecasting in enterprise systems. In: Conference on Knowledge Discovery in Data: Proceeding of the Eleventh ACM SIGKDD International Conference on Knowledge Discovery in Data Mining, 21–24 Aug. 2005. ACM, 2005, pp. 801–807. 10.1145/1081870.1081976.

[22] Tohka, J., and M. van Gils. Evaluation of machine learning algorithms for health and wellness applications: a tutorial. *Comput. Biol. Med.* 132:104324–104324, 2021. 10.1016/j.compbiomed.2021.104324. 33774270 10.1016/j.compbiomed.2021.104324

[23] Bouckaert, R. R., and E. Frank. Evaluating the replicability of significance tests for comparing learning algorithms. In: Advances in Knowledge Discovery and Data Mining. Lecture Notes in Computer Science. Berlin: Springer, 2004, pp. 3–12. 10.1007/978-3-540-24775-3_3.

[24] Lee, R., and W. F. Kean. Obesity and knee osteoarthritis. *Inflammopharmacology*. 20(2):53–58, 2012. 10.1007/s10787-011-0118-0. 22237485 10.1007/s10787-011-0118-0

[25] Sharma, L., J. Song, D. T. Felson, S. Cahue, E. Shamiyeh, and D. D. Dunlop. The role of knee alignment in disease progression and functional decline in knee osteoarthritis. *JAMA*. 286(2):188–195, 2001. 10.1001/jama.286.2.188. 11448282 10.1001/jama.286.2.188

[26] Vincent, K. R., B. P. Conrad, B. J. Fregly, and H. K. Vincent. The pathophysiology of osteoarthritis: a mechanical perspective on the knee joint. *PM & R*. 4(5):S3–S9, 2012. 10.1016/j.pmrj.2012.01.020. 22632700 10.1016/j.pmrj.2012.01.020 PMC3635670

[27] Ramazanian, T., S. Fu, S. Sohn, M. J. Taunton, and H. M. Kremers. Prediction models for knee osteoarthritis: review of current models and future directions. *Arch. Bone Joint Surg.* 11(1):1–11, 2023. 10.22038/ABJS.2022.58485.2897. 36793660 10.22038/ABJS.2022.58485.2897 PMC9903309

[28] Appleyard, T., M. J. Thomas, D. Antcliff, and G. Peat. Prediction models to estimate the future risk of osteoarthritis in the general population: a systematic review. *Arthritis Care Res. (2010)*. 2023. 10.1002/acr.25035. 10.1002/acr.25035 36205228

[29] Widén, E., et al. How communicating polygenic and clinical risk for atherosclerotic cardiovascular disease impacts health behavior: an observational follow-up study. *Circ. Genom. Precis. Med.* 15(2):e003459–e003459, 2022. 10.1161/CIRCGEN.121.003459. 35130028 10.1161/CIRCGEN.121.003459

