# Supplementary material for: Predicting Knee Osteoarthritis Severity from Radiographic Predictors: Data from the Osteoarthritis Initiative
Source: Ann Biomed Eng. 2025 May 9;53(8):1852–61. doi: 10.1007/s10439-025-03740-z (PMC12283875; doi:10.1007/s10439-025-03740-z)

**Supplementary**

**
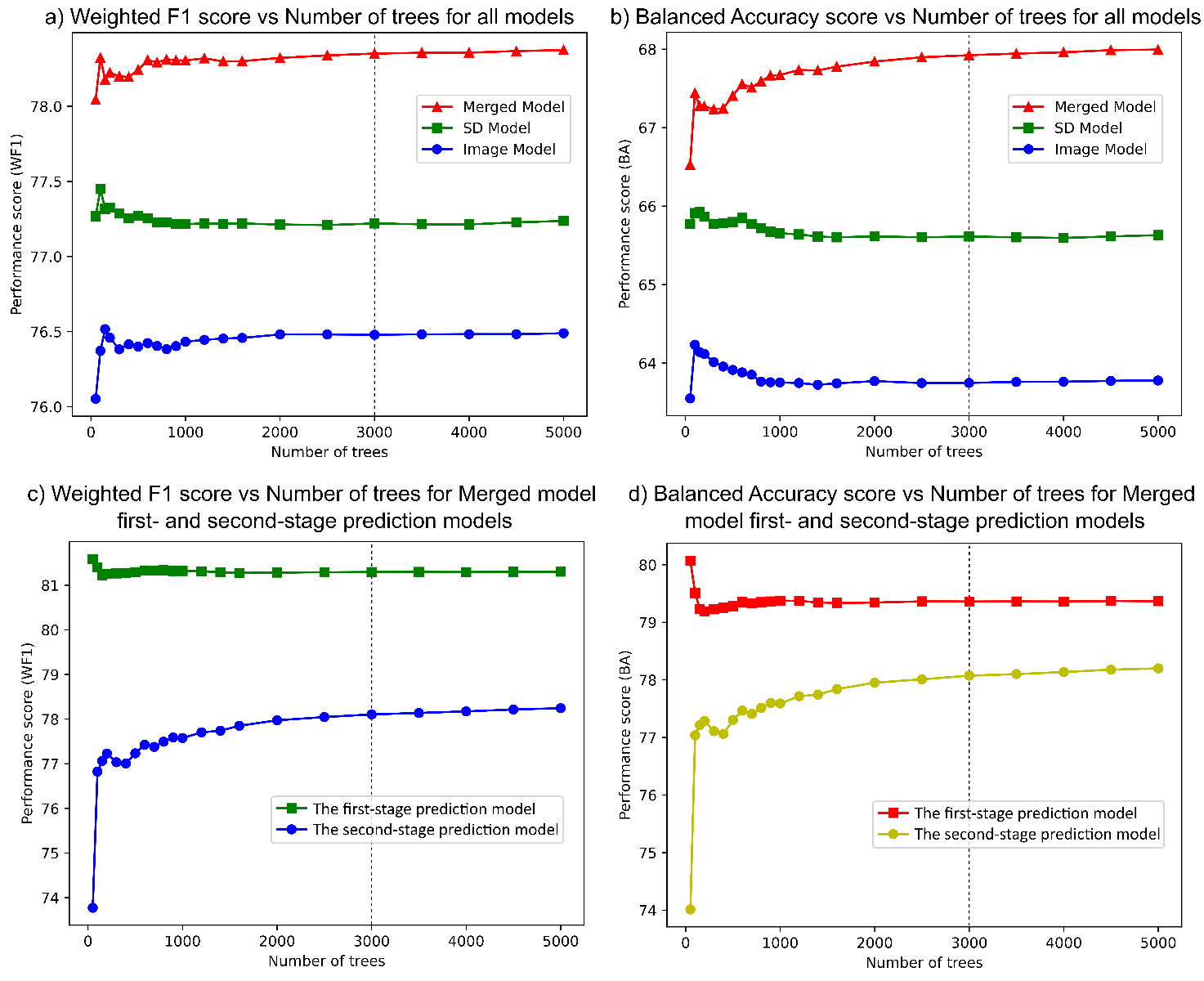
Figure S1:** a) Shows Weighted F1 (WF1) and b) balanced accuracy (BA) scores versus the number of trees used for training the model. c) Weighted F1 and d) balanced accuracy scores versus the number of trees used for training the Merged-model first and second prediction algorithms. We selected to use 3000 trees in the final models, which are highlighted in the graphs with dotted lines. Numbers of trees we tested were 50, 100, 150, 200, 300, 400, 500, 600, 700, 800, 900, 1000, 1200, 1400, 1600, 2000, 2500, 3000, 3500, 4000, 4500 and 5000.

**Figure S2:** Correlation matrix for all features listed in Table 1.

Correlation matrix


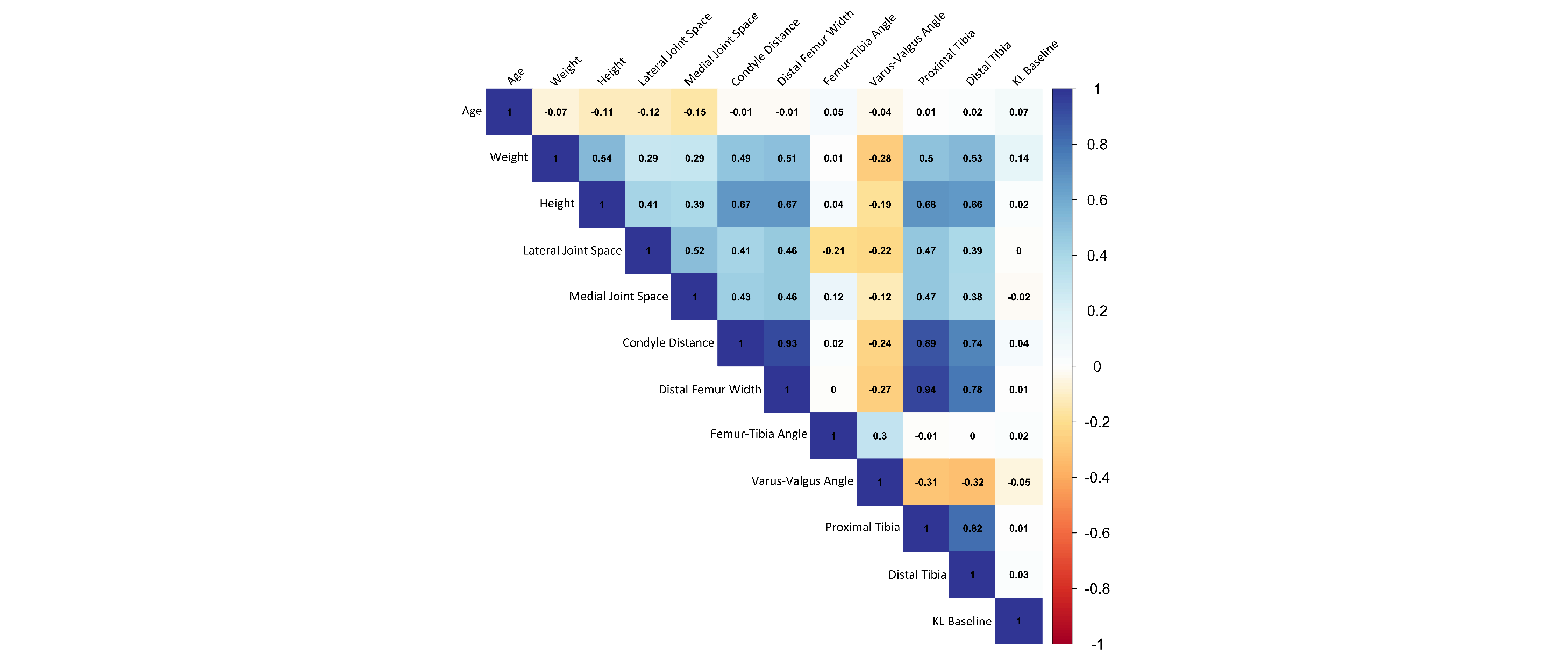

Supplement: Supplementary file 1 — Supplementary file1 (DOCX 322 kb) [file 10439_2025_3740_MOESM1_ESM.docx]
